# The COVID-19 Experience in Adolescents: Emotional and Behavioral Recall at the End of the Pandemic

**DOI:** 10.3390/diseases12060116

**Published:** 2024-06-02

**Authors:** Luciana Zaccagni, Federica De Luca, Natascia Rinaldo, Gianni Mazzoni, Simona Mandini, Emanuela Gualdi-Russo

**Affiliations:** 1 Department of Neuroscience and Rehabilitation, Faculty of Medicine, Pharmacy and Prevention, University of Ferrara, Corso Ercole I d’Este 32, 44121 Ferrara, Italy; luciana.zaccagni@unife.it (L.Z.); natascia.rinaldo@unife.it (N.R.); gianni.mazzoni@unife.it (G.M.); simona.mandini@unife.it (S.M.); 2 Center for Exercise Science and Sports, University of Ferrara, 44123 Ferrara, Italy

**Keywords:** COVID-19, adolescents, weight status, emotion, mental health, post-COVID

## Abstract

The COVID-19 pandemic and the resulting lockdown countermeasure may have significantly affected adolescents’ physical and mental health. This study aims to assess adolescents’ recollections of this period, also analyzing their current weight status along with factors they traced back to the epidemic phase and their current sports practice. A survey among 233 Italian adolescents aged 12.4 ± 0.9 years was conducted in October 2023. To achieve the research objectives, a new questionnaire was developed: the COVID-19 AdolesceNt/chilDren Lockdown Experience questionnaire (CANDLE). The new questionnaire was employed to gather data on the adolescents’ recollections of the lockdown situation they experienced. The stature and weight of participants were measured directly. The results indicated that middle schoolers remember both positive and negative experiences of the lockdown: the change perceived as the most positive was spending more time with family, while social detachment from peers represents the most negative aspect. According to multivariate regression analysis, certain behaviors they assumed during the lockdown, such as comfort food consumption in boys and sleeping disturbances in girls, in addition to their current sports practice, affected their actual Body Mass Index. This study supports the evidence that changes caused by the COVID-19 lockdown affected adolescents’ physical and mental health, albeit with sex differences.

## 1. Introduction

The recent COVID-19 pandemic, which started in China and spread rapidly around the world, severely affected all populations, not only because of the high morbidity and mortality rates, but also due to the effects of social isolation and distancing imposed by governments to stem the spread of the epidemic [1]. The main consequences of this on mental health have been depression, acute and post-traumatic stress, and anxiety [2]. Different symptoms were found during the COVID-19 outbreak depending on age, and in particular, increased anxiety and depression were reported in children aged 7 to 13 years. Greater inattentiveness and increased reassurance needs, in addition to poor academic performance, misconduct, depression, anxiety, and social isolation, were found in the most problematic cases [3]. In adolescents too, parents during the pandemic noticed anxiety, depression, misconduct, social isolation, low attention span, and impulsiveness [3]. Their level of depression and anxiety was higher than that shown by the children [4]. Above all, social isolation and loneliness would have led to an enhanced risk of anxiety and depression in children and adolescents, with an increased impact on mental health as the duration of loneliness increased [5]. During this period, the overuse of smartphones and Internet addiction were found to be associated with increased depression [6].

Moreover, the COVID-19 pandemic has increased sedentariness in children and adolescents due to restrictions on physical activity (PA) and sports, school closures, and reduced outdoor recreational opportunities [7,8]. A scoping review covering 150 studies conducted during the epidemic on children and youth confirmed decreased PA, more time spent on screens, and increased total sedentariness [9]. PA is related to good general health status and mental health. A questionnaire administered to Dutch children showed that pandemic isolation led to PA reduction and increased screen time at and after school closures [10]. More generally, the WHO’s recommendations [11] of at least 60 min of moderate to vigorous PA per day in the 5–17 age group could not be followed during that time. The restrictions introduced following the COVID-19 outbreak, breaking the daily habits of children and adolescents, caused modifications in their PA and eating behaviors: as well as a reduction in PA, there has been a general increase in food intake, particularly in junk food consumption, resulting in increased body weight [12,13].

In Italy, quarantine caused a significant decrease in total PA energy expenditure per week in individuals of all ages, with negative consequences on mental well-being [14,15].

As a result of the large number of people sick or deceased due to COVID-19, a complete lockdown occurred in Italy from 9 March to 3 May 2020. Italian schools closed due to the pandemic, starting from 24 February 2020 in the Emilia-Romagna region (from March 5 in the remaining part of Italy), and continued with e-learning until the end of the school year. All PA-related school activities and extracurricular sports or aggregate activities were simultaneously suspended. As a result, this negatively affected interpersonal relationships, resulting in emotional distress [16]. In a survey conducted three weeks after lockdown by the Gaslini Hospital in Genoa (Italy), the most frequent complaints of children and adolescents (6–18 years old) involved the “somatic component” (anxiety and psychosomatic complaints such as feeling short of air) and sleep disturbances (difficulty falling asleep and waking up to start e-learning at home) [17]. In older adolescents, increased emotional instability was also found, with irritability and changes in mood tone [17]. There was, however, a decrease in migraine symptoms during the lockdown due to lower stress related to school [18].

As schools reopened across the country, new restrictive rules on in-presence teaching activities were implemented in October 2020, from the use of face masks and hand sanitization to the scheduling of different entrances/timetables per class, and temperature control before the start of educational activities [19]. Nevertheless, the pandemic showed an increase in the rate of diagnosed cases in children and adolescents, from 1.8 percent (during the lockdown phase) to 8.5 percent. Most of the diagnosed cases occurred in adolescents aged 13–17 years (41.3%), followed by children aged 7–12 years (28.0%) and younger children [20]. Given this situation, subsequent Prime Ministerial Decrees, starting in November 2020, divided Italy’s regions according to the epidemiological situation, and a nationwide curfew was imposed between 10 p.m. and 5 a.m., in addition to banning all travel, closing shopping centers on weekends, and implementing distance education for high schools. Further decrees provided for adjustments to restrictive measures according to the epidemiological situation in the region. The state of emergency ceased in Italy on 1 April 2022. On 5 May 2023, the World Health Organization (WHO) declared the conclusion of the global health emergency caused by the Coronavirus Disease 2019 (COVID-19) [21,22].

Although many aspects related to the lack of PA and the mental health of children and adolescents during the epidemic period have been studied worldwide in the literature, little is known about the long-term effects and possible development of the related post-traumatic disorder [23]. The current study aims to fill this shortcoming by examining a sample of Italian adolescents who experienced the lockdown. Taking into account the more complex questionnaires previously developed regarding stress related to COVID-19 in adolescents (CASPE with 38 questions) [24] and the influence of lifestyle changes caused by the epidemic on mental health and behavior (CRISIS with 94 items) [25], we developed a new expedited post-COVID-19 questionnaire (COVID-19 AdolesceNt/chilDren Lockdown Experience questionnaire, CANDLE) for children and adolescents consisting of 11 simple questions related to their experience during the long-lasting lockdown in Italy.

Insights into adolescents’ physical and mental health in this post-COVID-19 phase may help to develop more informed and differentiated policies that can adapt to any new specific situation. We hypothesized that the resilience and mental health of children and adolescents enabled them to positively overcome their experiences during the outbreak caused by COVID-19 according to gender, and that misbehavior during the pandemic may have affected their actual weight status. Therefore, this study aimed to assess firstly participants’ emotions and lifestyle habits during COVID-19 by gender, and secondly how this may have affected their weight status.

## 2. Participants and Methods

### 2.1. Research Design, Procedure, and Participants

In this study, we adopted a cross-sectional design. The study received approval from the Ethics Committee of Area Vasta Emilia Centrale (CE-AVEC), Italy (approval code n. VT2022). This study is part of broader research focused on the growth and development of children and adolescents [26,27]. An a priori power analysis was performed to estimate the minimum sample size using the G*Power statistical program (version 3.1.9.6; Universitat Kiel, Kiel, Germany) for 80% power, medium effect size, and 0.05 significance level. The following minimum sample sizes were calculated according to Andrade [28] for the primary study hypotheses tested by gender comparisons: 128 participants for the *t*-test, and 122 participants for the chi-squared test. To this purpose, we invited all children from a middle school (chosen by convenience) in the city of Ferrara (Northern Italy) to participate in the study through an official letter from the school sent to families. All those who agreed to participate and had written, signed parental consent were considered in the study sample. Participation in the research was completely voluntary, and participants were assured of confidentiality and anonymity, with each form assigned a unique ID number. The sample thus consisted of 233 participants of both sexes (143 male and 90 female), aged 12.4 ± 0.9 years. Therefore, the minimum sample size assumptions of the power analysis are met in our study.

In October 2023, we collected data on the sample through direct anthropometric measurements and questionnaires. Specifically, before administering the questionnaire presented in this study, participants were asked some basic socio-demographic and sports-activity-related questions (yes/no; number of hours/week), and the anthropometric survey was conducted. Among anthropometric measurements, we measured stature using an anthropometer (Magnimeter, Raven Equipment Ltd., Dunmow, Essex, UK) and weight with a mechanical scale (SECA, Basel, Switzerland). A trained operator took anthropometric measurements for each participant according to standard anthropometric methods [29,30]. The body mass index (BMI), i.e., body weight (kg) divided by stature (m) squared, allowed us to classify participants based on Cole’s cut-offs [31,32] into the following categories: underweight, normal-weight, overweight, and obese.

Afterward, participants self-completed the new structured questionnaire (CANDLE), geared primarily toward assessing participants’ perceptions of the lockdown experience resulting from the COVID-19 outbreak. The CANDLE items can be traced as follows: i. COVID-19-related emotional experience, and ii. behavioral and lifestyle experience. In particular, the 11-item questionnaire was developed based on other questionnaires used during the COVID-19 epidemic (CRISIS, CASPE), the literature on the topic (among others, [1,33,34]), and discussions with experts. In particular, we considered issues listed in previous questionnaires, simplifying and reducing them numerically since CRISIS was aimed at adults, while CASPE partly addressed COVID-19 symptomatology and was, in any case, too detailed for early adolescents. Consistent with this approach, we reduced the number of possible answers per question to three. As for the literature consultation and discussion with the experts, these were crucial in confirming the importance of the topics to be submitted to the participants.

**Figure A1:**
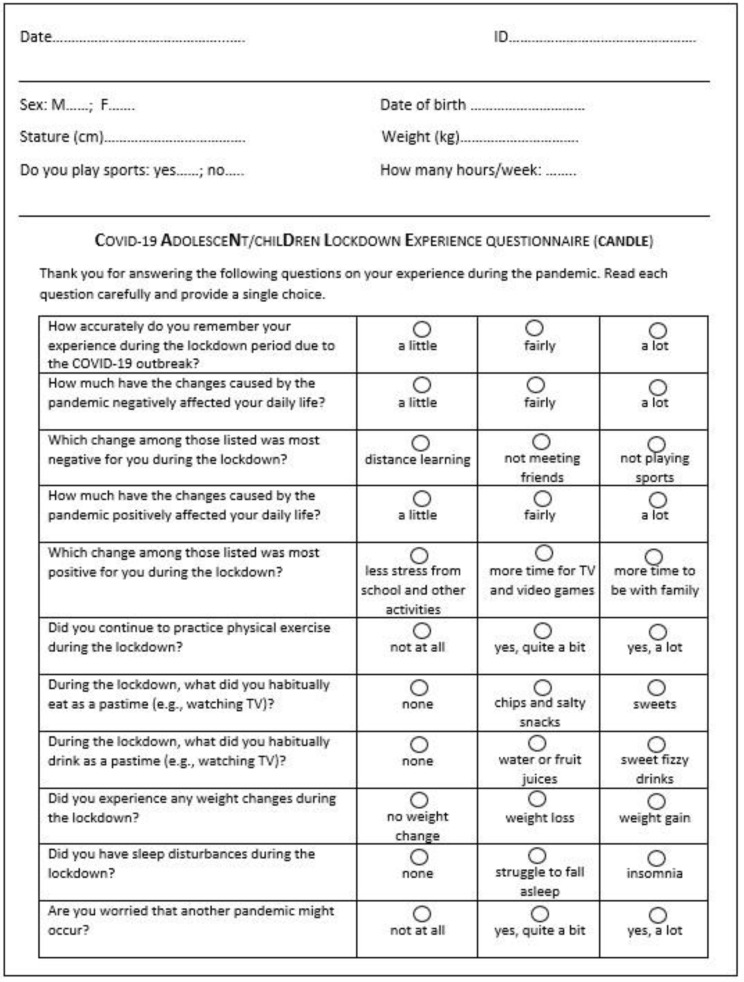
Data collection sheet.

Finally, we conducted a short pilot test, requesting some colleagues (n = 10) who were not engaged in the questionnaire development to ask their children to read and fill it out and give us feedback on its understandability and responsiveness. Based on the feedback, we made minor changes to the questionnaire to word the questions more clearly (Table 1). The questionnaire was created and submitted to participants in Italian. A copy of the complete data collection sheet with the questionnaire translated into English is shown in Figure A1 (Appendix A).

The stability of the CANDLE questionnaire over time was assessed using test–retest reliability at four weeks in a sub-sample of 21 students randomly selected from the total sample, who completed the same questionnaire on two different occasions. Pearson’s r coefficient was the statistic employed to measure test–retest reliability.

There were no missing values in anthropometric measurements or questionnaires collected in the survey.

### 2.2. Statistical Analysis

The reliability of the questionnaire was assessed using a test–retest method by calculating the correlation coefficients of the single answers to different questions. We used Pearson’s correlation coefficient to evaluate the strength and direction of the linear relationship between the scores obtained from two successive administrations of the same questionnaire. The correlation coefficient ranged between −1 and +1, where 0 denotes no correlation and 1 indicates complete correlation.

Descriptive statistics were used to summarize continuous variables (mean and standard deviation) and categorical variables (frequency and percentage). Differences between two independent groups (by gender) were evaluated using Student’s *t*-test, and the differences in frequency distribution between groups were assessed using the chi-squared test.

In addition to the possible direct influence of current sports practice, we examined which factors among those considered in this questionnaire influenced the participant’s current weight status (BMI), as there may be a possible indirect influence exerted by emotional factors and unhealthy habits related to the pandemic. Specifically, in addition to the weekly amount of current sports practice and age, we included all questions from the CANDLE questionnaire, except Q1, to determine their predictive power in a multiple regression analysis. After having checked the assumptions of multiple regression (independence, linearity, homoscedasticity, and normality), the analysis was conducted in backward mode in the two genders separately. The multicollinearity of the data was assessed through variance inflation factors (VIFs), taking VIF values in the range of 0.10 to 10 as acceptable [35].

For multiple regression analysis, the answer A1 was coded as “0” and the answers A2 and A3 were considered together and coded as “1” in questions Q2, Q4, Q7, Q10, and Q11. In Q3, Q5, and Q9, the answer A1 was codified as “1”, A2 as “2”, and A3 as “3”. Question Q6 was codified as follows: “1” for the first answer (A1) and “0” for the second or the third one (A2 and A3). In Q8, answers A1 or A2 were codified with “0” and A3 with “1”.

The data analysis was performed using Statistica for Windows, Version 11.0 (StatSoft Inc., Tulsa, OK, USA).

A *p*-value < 0.05 was regarded as statistically significant.

## 3. Results

The CANDLE questionnaire’s reliability was assessed using the test–retest method. Table 2 shows the correlation values obtained for the subsample of students (n = 21) who repeated the questionnaire 4 weeks later. Values of the r coefficient indicate moderate to strong correlation in repeated responses. The very high statistical significance (mostly *p* with a value of 0.001 or less) indicates the probability that the force expressed by r may arise by chance. In other words, the high *p* levels guarantee that correlations will be moderate or high (depending on r values) 99.99% of the time [36].

Table 3 displays anthropometric characteristics and information on the sports practices of the overall sample, and broken down by sex, at the time of data collection. As expected, boys and girls have similar mean values of stature and weight, and consequently BMI. Two-thirds of the sample stated that they practiced sport; the two sexes differed significantly in their sporting practice (73% in boys vs. 60% in girls; *p* = 0.0429), but reported a similar amount of weekly sports practice.

In the second part of the table, there is the sample distribution by weight status and sex. Boys and girls had a similar distribution (*p* = 0.8834): the most represented category was normal weight (over 65% in both sexes), one-twentieth of the sample was underweight, and over one-quarter was overweight or obese. In particular, in the overall sample, there was the same frequency of underweight and obese subjects.

Table 4 shows the frequency of the answers to the 11 questions of the CANDLE questionnaire by gender.

Most students of both genders had a fairly precise recollection of their experience during the lockdown due to the COVID-19 outbreak. The changes caused by the pandemic had few negative or positive effects on their daily lives; the most negative change was not meeting friends, and the most positive change was having more time to spend with family. Most male and female students continued to exercise during the lockdown, habitually ate chips and salty snacks, drank water or fruit juices as a pastime, and had no weight changes or sleep disorders.

The boys differed significantly from the girls in the question on exercise practice (Q6) during solitary confinement: almost a quarter of the boys (23.2%) answered ‘a lot’ versus only a tenth of the girls.

A different response frequency was also observed in the two genders concerning habitual eating pastimes (Q7): more than 40% of boys did not eat as a pastime compared to more than 30% of girls. Also, 16% of girls habitually ate sweets vs. 6% of boys. Another significantly different trend in the responses was observed in Q11 about the concern that another pandemic might occur: the majority of the boys (more than half) were not worried, while 74% of the girls said they were concerned to some extent.

Table 5 shows the results of multiple linear regression analyses for the current BMI values, as the dependent variable, and Q2, Q3, Q4, Q5, Q6, Q7, Q8, Q9, Q10, Q11, age, and weekly amount of sports practice as independent variables, separately by gender. Q7 was the only significant predictor in boys, while, in girls, Q10 and the weekly amount of sports practice were positively associated with the dependent variable.

The boys who ate nothing as a pastime during lockdown had a lower BMI than boys who ate chips and salty snacks or sweets. The girls who played more weekly sports at the time of data collection or who experienced no sleep disturbances during lockdown had a lower BMI than girls with sleep disturbances (struggling to fall asleep or insomnia). Both models are significant (*p* < 0.05) and account for 2.5% of the variance of BMI in boys and 18.6% (as indicated by R^2^ adjusted) in girls.

## 4. Discussion

During the COVID-19 pandemic, stress and the disruption of social relationships, including school attendance, certainly contributed to the onset of emotional disorders in adolescents, whereas inactivity and the consumption of unhealthy food may have led to an increased incidence of overweight and obesity. Although there have been several studies in the literature on these aspects carried out during the pandemic, it is crucial to detect what perceptions the adolescents retained of their experience after the pandemic was over, by distinguishing both the positive and negative effects these changes brought about in their lives. In this respect, our study is one of the first to set this aim, using a new purpose-built questionnaire (CANDLE) to assay potential risk and protective factors for adolescents’ emotional and physical health. The test–retest analysis generally showed large correlations, indicating that the CANDLE has acceptable to excellent reliability in 9 out of the 11 questions, with questionable reliability being apparent only for Q6 and Q8 [37]. However, it should be pointed out that we considered the test–retest only from the perspective of Pearson’s correlation, testing the degree of linear association between the two sets of scores and not their equality (agreement) [38].

The questionnaire developed is intentionally short, quick, and simple, both given the age of the respondents and to provide an overview of the emotions and behaviors that the adolescents retained as a memory of their pandemic experience. The very purpose of arriving at a user-friendly questionnaire tool led us to only 11 questions that concerned important information related to the recall of behaviors adopted during the lockdown and the emotions felt. Specifically, participants’ responses allowed us to assess the impact that the changes associated with the experience of COVID-19 had on adolescents’ lives and memories of their experiences. In addition, the analysis of BMI and weight status of the examined sample allows us to assess the possible influence exerted by some behaviors assumed during the pandemic, and the influence of the sports currently played. The study’s findings are noteworthy because they show that the experiences undergone during the lockdown were perceived to carry both positive and negative impacts on adolescents. A moderate/strong recollection of the experiences is still present in most of the surveyed sample: the change that for the majority weighed negatively on their lives concerned in both genders the disruption of daily social interactions with friends, while the increased time to be with family was judged a positive change by most boys and girls. A greater frequency in the habit of eating, especially sweets, as a pastime was found in girls. This factor, combined with the smaller number of girls who had maintained a high level of exercise during the pandemic, probably resulted in weight gain during this period, as they declared. Despite lower unhealthy food intake reported, the boys also recalled weight gain during this period. Again, PA was probably inadequate compared with their previous habits, although they exercised more than girls. At the time of this survey, the amount of exercising was significantly higher and undertaken for a greater number of hours per week in boys than in girls, consistent with the general trend of the phenomenon involving greater sports practice in males than in females [14,39].

Participants’ current weight status showed a prevalence of overweight/obesity of 29% in girls and 27% in boys, who also showed a larger number of normal-weight participants. We report these differences only as observed trends of the two sexes, as these were not statistically significant. It is likely that both sexes were probably negatively affected by their behaviors during the epidemic, as seems to be confirmed by the markedly lower percentage of obesity in the pre-COVID-19 period in adolescents aged 11–14 living in the Emilia-Romagna region, recorded as 4.7% in males and 1.5% in females according to a longitudinal study [40], with a presumed increase in obesity of 1.6% in boys and 2.9% in girls in the post-COVID-19 sample examined. More generally, a recent publication [41] reported worldwide changes from 1990 to 2022, with a “rise in obesity surpassing the decline in underweight”. Concerning children and adolescents, most countries show that obesity has more than doubled: the age-standardized prevalence of obesity in children and adolescents has increased in girls from 1.7% in 1990 to 6.9% in 2022 and in boys from 2.1% to 9.3%, with an increase in this interval of 51.2 million obese girls and 76.7 million obese boys [41]. Among the many reasons for this trend, the authors also recall the impact that COVID-19 may have recently had, as supported by a systematic review of longitudinal reports [42]. Notably, according to this review, there were small but significant increases in weight, BMI, and obesity prevalence in adults and, to a greater extent, in children in the first year of the pandemic. In essence, social isolation with the resulting lifestyle changes that increased energy intake over energy expenditure may be the main cause of the onset of obesity or its worsening, as well as its comorbidities, in children and adolescents [13,34]. Therefore, our findings, based mainly on participants’ recollections, are consistent with the above evidence and with other studies conducted during the pandemic that showed a significant decline in PA, an increased frequency of snacking, and weight increases in both adults [43,44] and children/adolescents [34,45].

Among the findings of the current study, those related to the negative effects caused by COVID-19 on various psychophysiological aspects of mental health should also be considered. Sleep disturbances during lockdown affected more girls (30%) than boys (24%). Concerning insomnia, girls have rates twice as high as boys, reaching the upper limit of the expected range for adolescents: according to the literature, 4–10% of adolescents experience symptoms of insomnia [46]. In addition to a trend of declining sleep hours observed over 20 years in adolescents, Keyes et al. confirmed differences in sleep by gender, with girls sleeping less. Although the mechanism is not entirely clear, these differences would depend on factors such as higher stress levels [47]. Other factors that have been highlighted include an increase in screen time, combined with a later bedtime, insomnia, or sleep difficulties, resulting in a decrease in sleep quality [46]. A greater difficulty in falling asleep and a tendency to delay bedtime and waking time during COVID-19 resulted in a comparison with pre-COVID-19 [48].

More than half of the male subsample, versus about a quarter of the female subsample, reported that they were not concerned about other possible future epidemics. Since hope and optimism are among the main constructs of resilience [49], the boys surveyed demonstrated greater resilience overall. The relevance of psychological resilience as a mental health determinant is well known [50]. The higher resilience shown by the male gender in this sample may have contributed to their improved sleep quality during the lockdown, compared to the female gender, through reduced anxiety and stress. It is well known from the literature that the female gender would appear to have suffered more mental health problems during the lockdown [51,52].

Multiple regression analysis controlled for age was applied separately by sex in this study to assess the relationships between post-COVID-19 BMI of participants and behaviors assumed during the epidemic, as well as current sports practice. We found that the influence of comfort food was the only predictor of current BMI in boys: lower current BMI values are shown in those who did not eat food as a pastime during lockdown compared to those who habitually ate it. In girls, the following two predictors of current BMI were found: sleep problems and actual sports hours/week. Girls who experienced sleep disturbances during the COVID-19 period and who are currently engaged in fewer hours of sports activity manifested higher BMI values. The association was greater in girls than in boys, explaining, respectively, a total variance of 19% and 2% in current BMI values. Both regression models show statistically significant *p*-values indicating a statistically significant relationship between predictors and the dependent variable (BMI). However, the low coefficient of determination (R^2^) obtained for the male gender may indicate that the influence of the selected predictor on BMI is rather weak, thus leading us to hypothesize an influence from other factors not considered in this study.

This study has some strengths: it reports retrospective data on adolescents’ perceived physical and mental health during the pandemic. The anthropometric data were directly collected by an expert anthropometrist. This is the first study reporting data 1.5 years after the end of the state of emergency in Italy and 5 months after the official end of the COVID-19 pandemic worldwide. The middle schoolers were recruited from a geographical area in northeastern Italy, located in the Po Valley, where the epidemic hit hard, and they were all subjected to the same restrictions for a lengthy time. The study design can be easily replicated, and information from further surveys can help clarify adolescents’ perceptions during the pandemic.

However, some limitations of the present study have also been considered. It should first be mentioned that the school was selected according to a convenience criterion, and, in addition, the study is mainly based on a self-administered questionnaire given to middle schoolers. Moreover, the questionnaire collects responses related to the lockdown phase, and adolescents may have faced some difficulties in remembering correctly. A further limitation is that the proposed questionnaire reports only qualitative data. Although important quantitative information (e.g., number of hours of sleep) is missing, this choice was dictated by the belief that quantitative data recalled by children/adolescents would have poor reliability, and it would be better to capture a general picture of the pandemic period that adolescents perceived and retained. In addition, since we do not have comparable pre-pandemic or current data for participants (except current data on sports practice or BMI), it is not possible to distinguish whether some of the behavioral factors (such as consumption of sugary drinks or junk food) identified here are really to be attributed to the pandemic context or are rather part of the participants’ lifestyle habits. There is no doubt, however, that the increased screen exposure time during the lockdown period fostered these practices [53]. Moreover, several studies have witnessed an increased consumption of unhealthy food and subsequent weight gain during the lockdown period [13,54].

## 5. Conclusions

CANDLE has proven to be a simple, practical, quick, and easily administrable questionnaire for adolescents aged 11–14 who faced lockdown during COVID-19. Because of its simplicity, the questionnaire can also be extended to children. Its possible further use, as well as in similar infectious disease outbreaks, can be expected in different situations where forced isolation is required. Although research on the effect of COVID-19 on adolescents’ mental and physical health and its influence in the near future is still in an up-and-coming stage, the results obtained in this study highlight the possible positive and negative outcomes of the COVID-19 pandemic as perceived by adolescents. Most participants perceived the pandemic-related changes positively, especially increased time spent with the family. However, significant gender differences were found, indicating a tendency toward greater anxiety in girls. Further studies need to be conducted to investigate variables related to the post-COVID-19 adolescent weight status and to understand the physical and mental health risks faced by adolescents who have experienced the pandemic. According to our results, unhealthy food, sleep disturbances, and a reduction/suspension of PA are risk elements recalled by participants. As these factors can affect future physical and mental health, interventions leading to monitoring and improving the health of post-pandemic adolescents are highly recommended.

## Figures and Tables

**Table 1:** CANDLE questionnaire item list.

Question	Answer 1	Answer 2	Answer 3
Q1. How accurately do you remember your experience during the lockdown period due to the COVID-19 outbreak?	a little	fairly	a lot
Q2. How much have the changes caused by the pandemic negatively affected your daily life?	a little	fairly	a lot
Q3. Which change among those listed was most negative for you during the lockdown?	distance learning	not meeting friends	not playing sports
Q4. How much have the changes caused by the pandemic positively affected your daily life?	a little	fairly	a lot
Q5. Which change among those listed was most positive for you during the lockdown?	less stress from school and other activities	more time for TV and video games	more time to be with family
Q6. Did you continue to practice physical exercise during the lockdown?	not at all	yes, quite a bit	yes, a lot
Q7. During the lockdown, what did you habitually eat as a pastime (e.g., watching TV)?	none	chips and salty snacks	sweets
Q8. During the lockdown, what did you habitually drink as a pastime (e.g., watching TV)?	none	water or fruit juices	sweet fizzy drinks
Q9. Did you experience any weight change during the lockdown?	no weight change	weight loss	weight gain
Q10. Did you have sleep disturbances during the lockdown?	none	struggle to fall asleep	insomnia
Q11. Are you worried that another pandemic might occur?	not at all	yes, quite a bit	yes, a lot

**Table 2:** Results of test–retest analysis for CANDLE questionnaire using Pearson’s r (n = 21).

Question	Correlation Value	*p*-Value
Q1	0.884	<0.001
Q2	0.695	<0.001
Q3	0.821	<0.001
Q4	0.738	<0.001
Q5	0.800	<0.001
Q6	0.646	0.001
Q7	0.856	<0.001
Q8	0.536	0.010
Q9	0.965	<0.001
Q10	0.659	<0.001
Q11	0.766	<0.001

**Table 3:** Anthropometric characteristics and sports practices of the sample (n = 233) by sex.

Variable	Overall n = 233	Boys n = 143	Girls n = 90	*p*-Value
Age (years)	12.4 (0.9)	12.4 (0.9)	12.3 (0.8)	0.2268 ^a^
Weight (kg)	48.8 (11.5)	48.7 (12.1)	49.0 (10.5)	0.8317 ^a^
Stature (cm)	155.3 (9.1)	155.6 (10.1)	154.9 (7.3)	0.5799 ^a^
BMI (kg/m^2^)	20.1 (3.6)	19.9 (3.6)	20.3 (3.6)	0.4050 ^a^
Sports practice (%)	67.8	72.7	60.0	0.0429 ^a^
Amount of sports practice (h/week)	4.3 (2.7)	4.5 (2.5)	3.8 (3.2)	0.1139 ^a^
Weight status:				0.8834 ^b^
Underweight	13 (5.6%)	8 (5.6%)	5 (5.6%)	
Normal weight	155 (66.5%)	96 (67.1%)	59 (65.6%)	
Overweight	52 (22.3%)	30 (21.0%)	22 (24.4%)	
Obese	13 (5.6%)	9 (6.3%)	4 (4.4%)	

^a^ Student’s *t*-test; ^b^ chi-squared test.

**Table 4:** Percent frequency distribution of answers to the questions of the CANDLE questionnaire by gender.

Question	Overall (n = 233)			Boys (n = 143)			Girls (n = 90)			*p*-Value ^b^
	A1	A2	A3	A1	A2	A3	A1	A2	A3	
Q1. How accurately do you remember your experience during the lockdown period due to the COVID-19 outbreak?	19.4	52.6	28.0	17.6	54.2	28.2	22.2	50.0	27.8	0.6719
Q2. How much have the changes caused by the pandemic negatively affected your daily life?	57.3	37.9	4.7	61.3	35.9	2.8	51.1	41.1	7.8	0.1199
Q3. Which change among those listed was most negative for you during the lockdown?	17.4	48.7	33.9	15.0	45.7	39.3	21.1	53.3	25.6	0.0874
Q4. How much have the changes caused by the pandemic positively affected your daily life?	53.0	38.8	8.2	52.8	38.7	8.5	53.3	38.9	7.8	0.9834
Q5. Which change among those listed was most positive for you during the lockdown?	37.5	16.4	46.1	39.9	16.8	43.4	33.3	15.7	50.0	0.5465
Q6. Did you continue to practice physical exercise during the lockdown?	33.2	48.7	18.1	30.3	46.5	23.2	37.8	52.2	10.0	0.0362
Q7. During the lockdown, what did you habitually eat as a pastime (e.g., watching TV)?	37.7	52.8	9.5	41.5	52.8	5.6	31.5	52.8	15.7	0.0255
Q8. During the lockdown, what did you habitually drink as a pastime (e.g., watching TV)?	10.0	76.6	13.4	9.9	74.5	15.6	10.0	80.0	10.0	0.4713
Q9. Did you experience any weight change during the lockdown?	49.1	18.5	32.3	49.0	17.5	33.6	49.4	20.2	30.3	0.8178
Q10. Did you have sleep disturbances during the lockdown?	73.8	19.3	6.9	76.2	18.9	4.9	70.0	20.0	10.0	0.2979
Q11. Are you worried that another pandemic might occur?	41.6	46.6	11.8	51.5	39.0	9.6	25.9	58.8	15.3	0.0009

A = answer; ^b^ chi-squared test.

**Table 5:** Predictors of BMI: results of multivariate regression analyses.

Variables	Boys			Girls		
	β	*p*-Value	VIF	β	*p*-Value	VIF
Q7 (no eating as a pastime)	−0.1790	0.0415	1.0000			
Q10 (no sleep problems)				−0.3610	0.0025	1.3156
Amount of sports practice (h/week)				−0.2446	0.0420	1.3848
R^2^	0.0321			0.3468		
R^2^ adjusted	0.0245			0.1860		
*p*-value	0.0415			0.0156		

β: Standardized regression coefficient; VIF = variance inflation factor; R^2^: coefficient of determination; R^2^ adjusted: coefficient of determination adjusted for the number of predictors in the model.

## Data Availability

Data are available upon request due to ethical restrictions regarding participant privacy. Requests for the data may be sent to the corresponding authors.
